# Optimization of Silicon Nitride Nanopowder Content in Polyamide 12 (PA12) in Extrusion-Based Additive Manufacturing

**DOI:** 10.3390/nano16010047

**Published:** 2025-12-29

**Authors:** Markos Petousis, Apostolos Korlos, Nikolaos Michailidis, Vassilis M. Papadakis, Apostolos Argyros, Nikolaos Mountakis, Maria Spyridaki, Athena Maniadi, Amalia Moutsopoulou, Nectarios Vidakis

**Affiliations:** 1 Department of Mechanical Engineering, Hellenic Mediterranean University, 71410 Heraklion, Greece; markospetousis@hmu.gr (M.P.); mountakis@hmu.gr (N.M.); mspyridaki@hmu.gr (M.S.); maniadi@materials.uoc.gr (A.M.); amalia@hmu.gr (A.M.); 2 Department of Industrial Engineering and Management, International Hellenic University, 14th km Thessaloniki—N. Moudania, Thermi, 57001 Thessaloniki, Greece; apkorlos@ihu.gr; 3 Physical Metallurgy Laboratory, Mechanical Engineering Department, School of Engineering, Aristotle University of Thessaloniki, 54124 Thessaloniki, Greece; nmichail@auth.gr (N.M.); aargyros@auth.gr (A.A.); 4 Centre for Research & Development of Advanced Materials (CERDAM), Balkan Centre, Building B’, 10th km, Thessaloniki-Thermi Road, 57001 Thessaloniki, Greece; 5 Department of Industrial Design and Production Engineering, University of West Attica, 12243 Athens, Greece; v.papadakis@uniwa.gr; 6 Department of Materials Science and Technology, University of Crete, 70013 Heraklion, Greece

**Keywords:** polyamide 12 (PA12), silicon nitride (Si_3_N_4_), additive manufacturing (AM), three-dimensional (3D) printing, nanocomposites, ceramics

## Abstract

The use of polyamide-12 (PA12) thermoplastics in additive manufacturing (AM) is promising owing to their mechanical properties and printability. However, in load-bearing applications, improvements in mechanical strength and stiffness are sought after. Herein, the reinforcement efficiency of silicon nitride (Si_3_N_4_) nanoparticles in the PA12 matrix was explored. The filler loading varied between 2.0 wt. % and 10.0 wt. %. The nanocomposites were extruded into filament using melt compounding for subsequent material extrusion (MEX) 3D printing. PA12/Si_3_N_4_ nanocomposites were examined for their thermal, rheological, morphological, and structural characteristics. For mechanical characterization, flexural, tensile, microhardness, and Charpy impact data were obtained. For structural examination, porosity and dimensional deviation were assessed. Scanning electron microscopy (SEM) was used to investigate morphology and chemical composition. The results indicate that Si_3_N_4_ nanopowder significantly improved all mechanical properties, with a greater than 20% increase in tensile strength and elastic modulus when compared to neat PA12. The structural characteristics were also improved. These findings indicate that Si_3_N_4_ nanoparticles provide a viable reinforcement filler for PA12 for use in lightweight, robust structural components fabricated using MEX AM. Furthermore, it can be stated that ceramic–polymer nanocomposites further improve the applicability of PA12, where high mechanical performance is required.

## 1. Introduction

The innovative manufacturing process [1] of additive manufacturing (AM) can be beneficial for material waste minimization, production time reduction, and design flexibility enhancement [2]. Thus, traditional manufacturing methods that operate with either material removal or shaping can be replaced [3], depending on the case. Industries exploiting AM include healthcare [4,5], automotive [6,7], and aerospace [8,9] because of the precision and geometric structure complexity provided during the layer-by-layer deposition of materials [10]. The design of each item built via AM is based on a 3D geometric model [11].

Different AM techniques possess unique characteristics and principles of operation [12,13]. In addition, there is a great variety of polymers utilized in AM, which can be classified into three different groups [14]: those for general purposes (e.g., Polylactic Acid (PLA) [15,16], acrylonitrile butadiene styrene—ABS [17,18], and polyethylene terephthalate glycol—PETG [19,20]), engineering grade (e.g., acrylonitrile styrene acrylate—ASA [21], polyamide [22,23], polycarbonate—PC [24,25], high-density polyethylene—HDPE [26]), high-performance (e.g., polyvinylidene fluoride—PVDF [27,28], polyether ether ketone—PEEK [29,30], and polysulfone—PSU [31]), and ultra-performance grade [32] (e.g., polyetherimide—PEI [33,34], polyimide—PI [35], and polyphenylene sulfide—PPS [36,37]).

The semi-crystalline synthetic polymer polyamide (PA) belongs to the family of thermoplastics and includes different grades [38]. Depending on the number of carbon atoms in each nylon monomer, different nylons are named [38], such as polyamide 66 (PA66), PA6, and PA12 [38]. They are extensively utilized in research and applications related to biomedical issues [39] (tissue engineering [40], drug delivery [41], prosthetics [42], and dental care [43]) and the automotive industry [44,45]. Specifically, PA12 is known to exhibit excellent mechanical behavior, thermal stability, chemical resistance, low porosity, and manufacturing flexibility [2,46]. Its low moisture absorption makes it a preferred choice for the transportation industry over other polyamides [47]. In 3D printing, it has been thoroughly investigated for its mechanical performance [39,48,49] and as a matrix for composite development using ceramic [50], carbon [2,51,52], or other additives [53].

PA12 interest is expected to grow gradually in response to application demands [54,55]. It is stated by Fundamental Business Insights that the PA12 market size is expected to increase from USD 1.49 billion to USD 2.47 billion from 2025 to 2034, achieving a compound annual growth rate—CAGR greater than 5.2% (base year 2024 with USD 1.49 billion) [56]. Moreover, Verified Market Reports indicated an estimation of USD 1.5 billion in 2024, expected to increase up to USD 2.5 billion by 2033 (6.5% CAGR) [57]. An even greater CAGR was estimated by Zion Market Research to be expected between the years 2024 and 2034, increasing from USD 2.05 billion to USD 4.60 billion [58].

Although polymers can be used for a wide variety of purposes, there are still requirements of the manufacturing industry, mainly related to their mechanical performance, which cannot be satisfied [59]. To address this issue, a wide range of additives is available to provide reinforcing properties and characteristics depending on each application requirement through the development of composites and nanocomposites. Reinforcements such as glass [60], carbon [61,62], natural [63,64], or boron [65,66] fibers, as well as metallic [67] (Al [68]), ceramic [69] (silicon nitride—Si_3_N_4_ [70]), organic (cellulose [71,72]), and carbon-based [73] (graphene [74]) fibers, have been utilized.

Synthetic non-oxide ceramics of silicon nitride [75] possess excellent mechanical, thermomechanical, and tribological properties, in addition to being biocompatible [76]. It is a ceramic material that can be utilized in aerospace [77], biomedical [78,79,80], and other applications [81]. In the available literature, they have been combined with polymers as reinforcement [82] and processed via 3D printing to study the performance of materials such as PLA [83,84], polypropylene (PP) [85], PETG [86], HDPE [87], biomedical resin [88], ASA [89], ABS [90], and even high-performance PEEK thermoplastics [91,92]. PLA/Si_3_N_4_ composites with varying filler amounts were prepared to assess their mechanical and thermal properties. This study demonstrated similar reinforcing effects of the addition of Si_3_N_4_ on the tensile, flexural, compressive, and impact properties. The 5 wt. % Si_3_N_4_ composite featured a reduction in porosity, which is consistent with the findings of this study [83]. Table 1 summarizes a comparison between the improvements in the main properties achieved by the addition of Si_3_N_4_ nanocomposites to various matrices. The same grade of nanoparticles was used in this study. Material extrusion (MEX) and vat photopolymerization nanocomposites were presented. As shown, the reinforcing effect is similar between the polymers presented, and in many cases, the optimum filler loading was the same as that in the current research. Higher differences were observed in the stiffness improvement, with PLA [84] having a greater reinforcement effect and ASA [89] being lower than those of the other polymers. Such differences justify the need for studying each polymer individually and each preparation method, as the interaction between the additive and the matrix leads to different results regarding the improvement in mechanical performance.

Considering the growth of the silicon nitride market, Research Nester reported a 7.5% CAGR between 2026 and 2035, increasing from USD 135.79 million to USD 262.16 million [93]. Previous research reported an estimated CAGR of 8.37% between 2025 and 2034, by achieving an increase from USD 59.55 million to USD 122.73 million [94]. An estimation reported by Future Market Insights about the forecast period of 2025–2035, indicated an Si_3_N_4_ market size rise from USD 145.7 million to USD 289.2 million (7.1% CAGR) [95]. In addition, similar reports can add evidence to the silicon nitride market size increase and prove its usefulness in various sectors [96,97].

The PA12 polymer and Si_3_N_4_ additive were selected as the subjects of this research as the matrix and filler materials, respectively. Initially, the materials for the compositions of the five different mixtures, i.e., 2.0 wt. %–10.0 wt. % with a gradual filler loading increase of 2.0 wt. %. The mixtures were converted into extruded filaments and filaments into 3D-printed specimens in three different forms: tensile, flexural, and Charpy impact tests. Apart from the mechanical tests conducted in this study, the investigation consisted of various analyses and examinations. Thermogravimetric analysis (TGA) and differential scanning calorimetry (DSC) were used to analyze the thermal characteristics, while viscosity and melt flow rate (MFR) were used to determine the rheological characteristics. Moreover, Raman spectroscopy was carried out along with scanning electron microscopy (SEM) to determine the morphological features, and micro-computed tomography (μ-CT) was used to determine the structural (quality-related) properties. The most remarkable performance was exhibited by PA12/6.0 wt. % Si_3_N_4_, which revealed an improvement for the majority of mechanical properties and the structural characteristics as well, in relation to pure PA12. In brief, this research aims to investigate the reinforcing abilities of Si_3_N_4_ on the performance of PA12 polymers from the mechanical and morphological points of view. The aim was to introduce novel nanocomposites for MEX AM and discover their potential utilization of such PA12/Si_3_N_4_ nanocomposites, for future applications based on their respective requirements. Finally, by gradually covering the research gaps, we aim to lead the way to new unexplored paths for polymers and their presence in 3D printing-related industries.

The novelty of this research is related to its rigorous multiscale analysis of PA12/Si_3_N_4_ nanocomposites, encompassing a substantial filler range of 2.0 wt. %–10 wt. %, within the context of MEX AM. In this technological domain, the interactions between coupled ceramic and polymer components remain inadequately understood. This study introduces a comprehensive framework to systematically correlate nanoparticle loading with thermal–mechanical transitions, structural characteristics, interfacial bonding mechanisms, and, ultimately, the quality of the final part in MEX-printed PA12. It also reveals the threshold behavior at loading levels, providing new insights into printability limits and the microstructural origins of the gains or drawbacks. This study advances the scientific understanding of ceramic-reinforced polymer nanocomposites in extrusion additive manufacturing and contributes to the design principles for the emerging field of next-generation high-performance PA12 nanocomposites.

## 2. Materials and Methods

**Figure 1:**
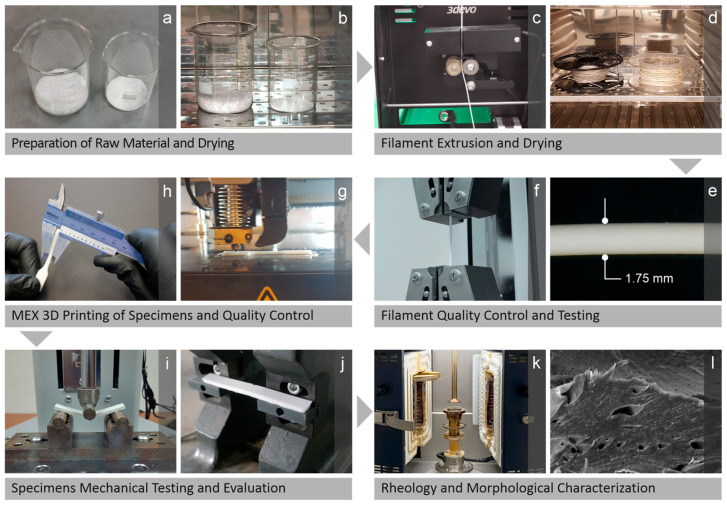
Research work steps: (**a**) PA12 and Si_3_N_4_ raw material preparation and (**b**) oven drying, (**c**) extrusion of the respective filaments and their (**d**) oven drying (moisture removal), (**e**) quality control (filament) (**f**) experiments, (**g**) specimen fabrication via MEX 3D-P and their (**h**) manual measurements on tested samples, (**i**–**l**) mechanical testing as well as evaluation, and (**k**) rheological characterization and (**l**) morphological inspection.

The two materials supplied for this research work conducted herein, namely PA12 as the matrix polymer and Si_3_N_4_ as the ceramic additive, were initially prepared while still being in their raw form and then placed in a laboratory oven for dehydration (Figure 1a,b). The resulting PA12/Si_3_N_4_ mixtures (individual for each loading) were then supplied to an extruder to be properly composed and converted into filaments, which were also placed in the oven to remove moisture (Figure 1c,d). The filaments were later tested and subjected to quality control (Figure 1e,f) before fabrication with MEX 3D printing of the coupons (Figure 1g). The produced specimens were inspected for quality (Figure 1h) before mechanical testing and evaluation (Figure 1i,j). Their rheology and morphology were examined using the respective methods and technologies (Figure 1k,l).

### 2.1. Materials

Polyamide 12 (PA12) in the form of fine grains was purchased from Arkema S.A. (grade Rilsamid/PA12/AESNOTL; the company is located in Colombe, France). As stated in the respective datasheets, the melt volume flow rate was 8 cm^3^/10 min (ISO1133). Silicon nitride (Si_3_N_4_) nanoparticles were purchased from Nanographi, Ankara, Turkey. These nanoparticles feature (based on their manufacturer information) Purity: 99.6%, Size: 760 nm, and contain Si, Cl, and O in their chemical composition.

### 2.2. PA12/Si3N4 Filament Extrusion and Specimen 3D Printing

The initial step for the PA12/Si_3_N_4_ nanocomposites to be created was to measure the necessary quantities based on the filler percentages under investigation, namely 2.0 wt. %–10.0 wt. % (2.0 step). The additive loading selection was decided based on the findings of preliminary experiments on coupons with increasing nanoparticle quantity, which was terminated by the time the sample performance declined in successive loading quantities. The resulting mixtures were dried in a laboratory oven at 80 °C for 8 h. A 3D Evo Composer 450 extruder (equipped with a single screw by 3devo, B. V., Utrecht, The Netherlands) was utilized for filament extrusion. Extrusion monitoring was also performed to ensure that the filament was in a 1.65 mm–1.85 mm diameter range. If the deviation in the diameter of the produced filament was higher, micro-adjustments were made to the extrusion settings. The filament fabrication parameter selection was based on information available in the literature [85]. The extruding conditions were as follows: zone 1220 °C (nozzle); zone 2230 °C; zone 3230 °C; zone 4200 °C (hopper); and an extruder speed of 4 rpm.

Filament drying overnight in a laboratory oven at 80 °C preceded them being fed to a Fused Filament Fabrication (FFF) 3D printer by the company Intamsys, model Funmat HT (Intamsys Technology Co., Ltd., Shanghai, China), to manufacture the 3D-printed specimens. The Fusion 360™ computer-aided design platform (Autodesk^®^ Inc., San Francisco, CA, USA) was used to design the desired specimen models.

### 2.3. Morphological Characterization and Energy Dispersive Spectroscopy

Three-dimensionally printed specimens were examined using SEM, which captured pictures of their fractured and lateral surfaces at different magnifications. A field-emission SEM microscope (Jeol, Tokyo, Japan), model JSM-IT700HR, was used at an acceleration voltage of 20 kV, operated in high-vacuum mode, and gold-sputtered samples were observed. The same apparatus was used for energy dispersive spectroscopy (EDS), which revealed the elemental composition of the nanocomposites and dispersion of the nanoparticles in the observed areas.

**Figure 2:**
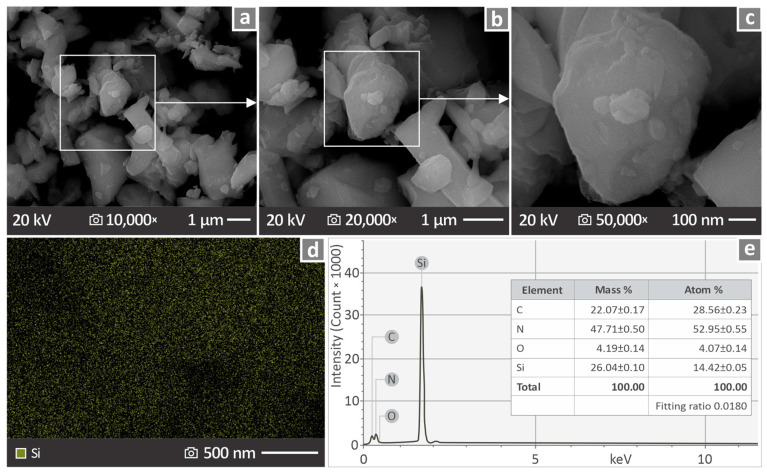
Si_3_N_4_ nanoparticles: (**a**–**c**) SEM pictures in magnifications of 10,000×, 20,000×, and 50,000×, (**d**) EDS mapping showing Si dispersion, and (**e**) EDS elemental composition graph.

The same apparatus was used to preliminarily examine the nanoparticles for their shape and size of the NPs. In Figure 2a–c, Si_3_N_4_ nanoparticles are depicted and captured via SEM. Observation regions were magnified 10,000×, 20,000×, and 50,000×, each succeeding the previous one in a greater magnification of the region marked by the square. In addition, the EDS mapping of Si_3_N_4_ nanoparticles is presented in Figure 2d, revealing the Si element dispersion, while Figure 2e shows an EDS graph that includes a full chemical composition analysis. As anticipated, high levels of Si were observed.

### 2.4. Mechanical Characterization

To examine the PA12/Si_3_N_4_ mechanical performance, a series of experiments was performed with respect to their tensile, flexural, and Charpy impact behaviors and microhardness. The devices utilized for this purpose and the corresponding international standards complied with by the research experiments are as follows:

- Tension testing was performed on V-type coupons with a thickness of 3.2 mm, in accordance with the standard ASTM D638-02a. The apparatus used was an MX2 motorized testing stand by Imada (Imada Inc., Tokyo, Japan) featuring two uniform grips in tensile operation (elongation was set at 10 mm/min).
- Flexure 3-point test, according to ASTM D790-10. The same motorized testing stand was utilized with an appropriate setup, featuring a 52.0 mm clearance (elongation was set at 10 mm/min).
- Impact, following ASTM D6110-04, utilizing a Charpy impact apparatus by the Terco company (Terco, Kungens Kurva, Sweden) model named MT 220 (367 mm hammer release height [98]).
- The microhardness of the fully polished specimens, following ASTM E384-17, was measured using an apparatus manufactured by Innova Test, model Vickers 300 (Maastricht, The Netherlands) device, with an applied force of 100 gF for an indentation duration of 10 s.

**Figure 3:**
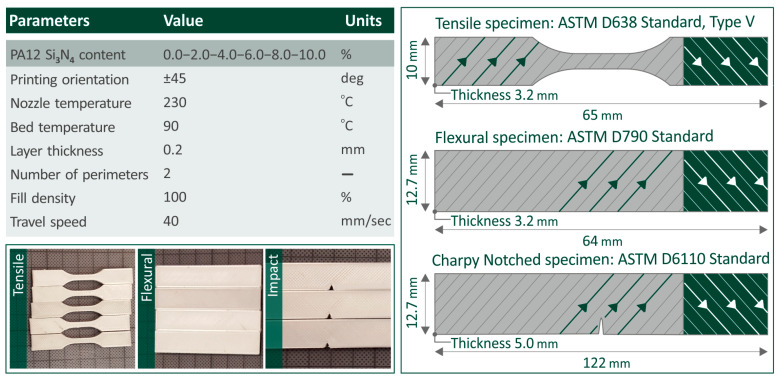
List of the specimen 3D printing parameters, models, and dimensions, as well as images captured of the flexural, tensile, and impact 3D-printed coupons. The 3D printing raster orientation is presented with diagonal lines and arrows (±45° angle, changing in sequential layers, to decrease anisotropy in the structure).

The utilized 3D printing parameters and models of the specimens are provided in Figure 3 along with images of some fabricated tensile, flexural, and Charpy impact samples. In accordance with the standards, five samples were tested per case (filler loading) for each mechanical experiment. All experiments were performed under ambient conditions (temperature and humidity).

### 2.5. Raman Spectroscopy and Thermal, Rheological, and Structural Characterizations

- The Raman spectra were obtained using a Raman Spectrometer LabRAM HR from the HORIBA Scientific company, based in the city of Kyoto, Japan.
- TGA was implemented on a Diamond Perkin Elmer (Shelton, CT, USA) apparatus.
- DSC was performed using a model DSC-25 Discovery Series (TA Instruments, New Castle, DE, USA).
- Viscosity and MFR measurements were taken with a Discovery Hybrid Rotational Rheometer DHR-20 Series (TA Instruments, DE, USA).
- The dimensional deviation and 3D printing structure porosity were evaluated using a Compact 225 kV Tomoscope HV Micro Focus CT scanner (Werth Messtechnik GmbH, Giessen, Germany).

The methodology followed is provided in the Supplementary File.

## 3. Results

### 3.1. Thermal Properties

**Figure 4:**
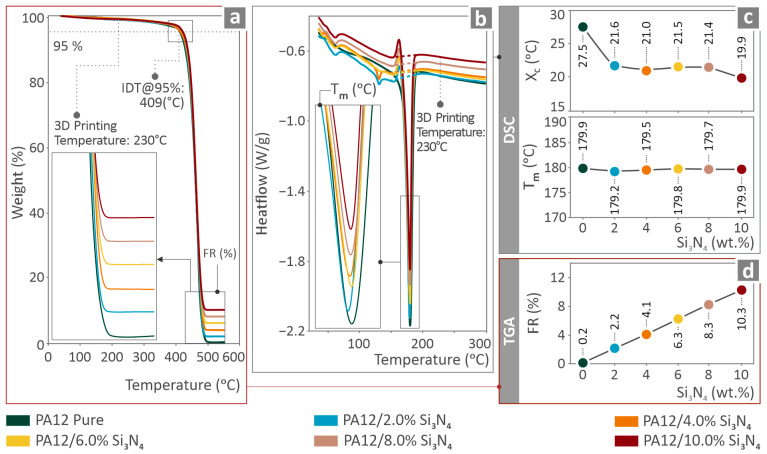
PA12/Si_3_N_4_ samples: (**a**) TGA graph, (**b**) DSC graph, (**c**) *X_c_* and T_m_ values derived with DSC, and (**d**) final residue values derived with TGA.

Figure 4 shows the TGA and DSC results for the PA12/Si_3_N_4_ samples. Figure 4a shows the weight as a temperature graph (TGA). Acute decomposition started at approximately 409 °C, whereas the printing temperature was 230 °C. Thus, no degradation was expected to occur during processing, which would have an impact on the mechanical behavior [98]. Furthermore, the residual increased with filler loading, which was the expected outcome (Figure 4d). Figure 4b shows the heat flow compared with the temperature graph (DSC). The *X_c_* and T_m_ values are shown in Figure 4c. Three-dimensional printing temperature was approximately 50 °C higher than the T_m_ values. As the literature instructs [99], effective extrusion requires the melt temperature to exceed T_m_, beyond the crystalline melting range, not simply at the DSC peak T_m_. Otherwise, the polymer may not be fully molten, leading to poor flow, high viscosity, incomplete fusion, and, as a result, weak mechanical properties. The DSC findings are summarized in Table 2.

### 3.2. Raman Spectroscopy Results

**Figure 5:**
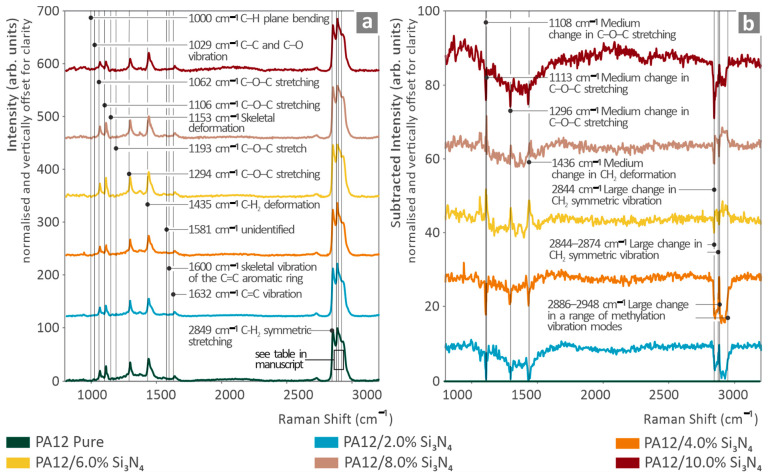
(**a**) Raman spectra for all samples tested; (**b**) Raman spectral differences of nanocomposites, compared to unfilled PA12.

Figure 5a shows the Raman spectra of pure PA12 and PA12/Si_3_N_4_ nanocomposites. The Raman peaks of the pure PA12 coupon were detected and assessed using the literature [102,103,104,105,106,107,108,109]. These are shown in the Supplementary File in a table form (Table S1).

The progressive addition of Si_3_N_4_ resulted in changes in Raman signals. The Raman lines (1108, 1113, and 2844 cm^−1^) exhibited intensity changes that were linearly related to the concentration increase as the Si_3_N_4_ wt. % increased. In the Raman lines at 1296 and 1436 cm^−1,^ there were intensity changes, but there was no clear relation to the increase in the concentration of Si_3_N_4_. The aforementioned changes can be seen in Figure 5b, where, although the concentration of Si_3_N_4_ increases in PA12, the respective Raman lines of pure PA12 are differentiated in intensity.

The addition of Si_3_N_4_ to PA12 resulted in an intensity drop in the Raman profile at 1108 cm^−1^ (change in C-O-C stretching [102]), 1113 cm^−1^, and 2844 cm^−1^. An intensity change with no clear relationship to the Si_3_N_4_ concentration increase was observed at 1296 cm^−1^ (skeletal stretching, C-O-C bonds [103,105]) and 1436 cm^−1^ (C-H_3_ deformation [102,105]; C-H_2_ deformation [102,105]; and C-H_3_ symmetric bending [102,104,110]). Lastly, a broad intensity drop appears in the ranges of 2844–2874 cm^−1^ and 2886–2948 cm^−1^ related to changes in the methylation vibration mode range [103]. The data are presented in Table 3.

### 3.3. Rheology Data

**Figure 6:**
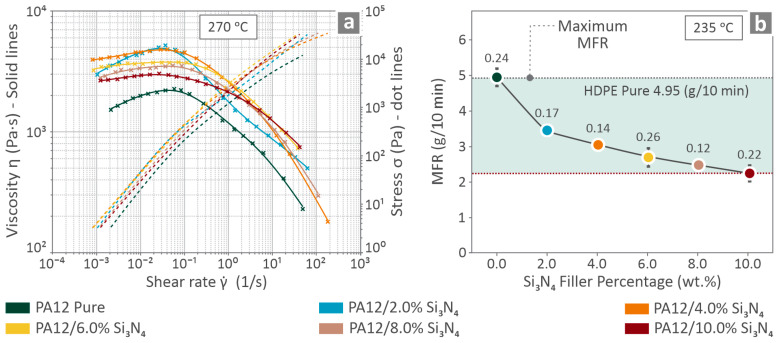
PA12/(0.0 wt. %–10.0 wt. %) Si_3_N_4_ rheological characteristics, namely (**a**) viscosity and stress curves versus shear rate and (**b**) MFR levels (the numbers on top of the average values presented in the graph are the standard deviations calculated in each case).

The rheological characteristics of the PA12/(0.0 wt. %–10.0 wt. %) Si_3_N_4_ composites and pure PA12 are presented by Figure 6. Viscosity and stress curves compared to the shear rate (at 270 °C) are shown in Figure 6a, indicating that the viscosity tends to decrease as the stress increases. The addition of nanoparticles increased viscosity. The MFR (at 235 °C) related information can be found in Figure 6b, where it is revealed that MAFR reduces as the filler percentage increases, which is also an indication of viscosity increase.

### 3.4. Quality Control and Mechanical Testing of the Extruded Filament

**Figure 7:**
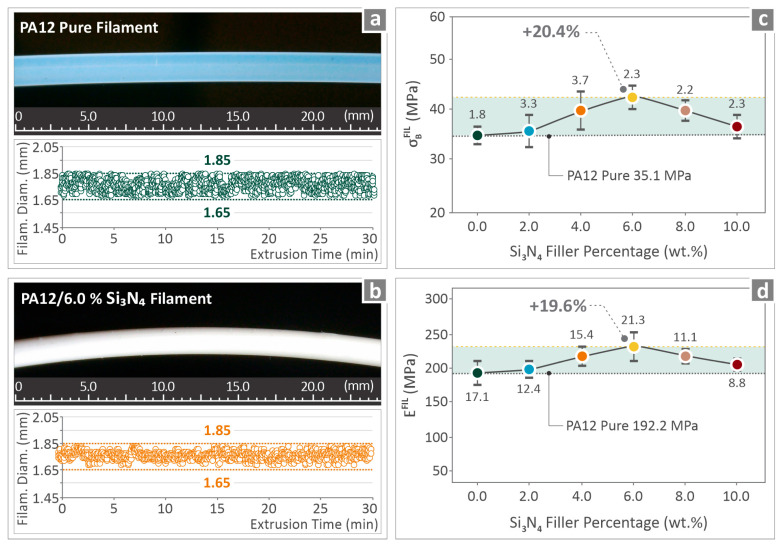
(**a**,**b**) Pure PA12 and PA12/6.0 wt. % Si_3_N_4_ filament section images and diameter monitoring results; (**c**,**d**) PA12/(0.0 wt. %–10.0 wt. %) Si_3_N_4_ filament tensile strength and Young’s modulus levels. Different colors depict the different loadings in the nanocomposites.

Quality control for pure PA12 and PA12/6.0 wt. % Si_3_N_4_ is presented in Figure 7a,b. The filament images and diameter monitoring results are presented. Both seem to be of great quality, with a diameter within acceptable margins. Figure 7c,d show the strength in the tensile test along with the stiffness (modulus of elasticity) levels measured for the tested samples of PA12/(0.0 wt. %–10.0 wt. %) Si_3_N_4_ nanocomposites. For both the properties, a clear improvement was observed as the filler percentage increases to 6.0 wt. %, which was measured to possess the highest levels, increased by 20.4% and 19.6% respectively, over pure PA12. The two remaining fillers after 6.0 wt. % (8.0 wt. % and 10.0 wt. %) began to decrease, although they remained over pure PA12 levels.

### 3.5. Mechanical Properties

**Figure 8:**
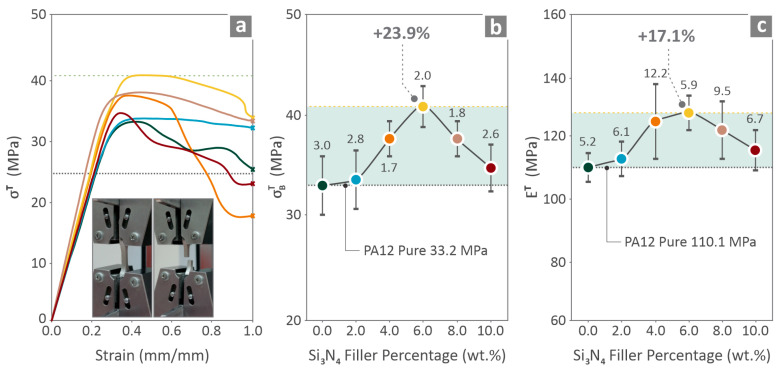
PA12/(0.0 wt. %–10.0 wt. %) Si_3_N_4_ tension mechanical data: (**a**) stress vs. strain curves and tensile testing image; (**b**) strength vs. Si_3_N_4%_ and (**c**) modulus of elasticity vs. Si_3_N_4_. Different colors depict the different loadings in the nanocomposites.

**Figure 9:**
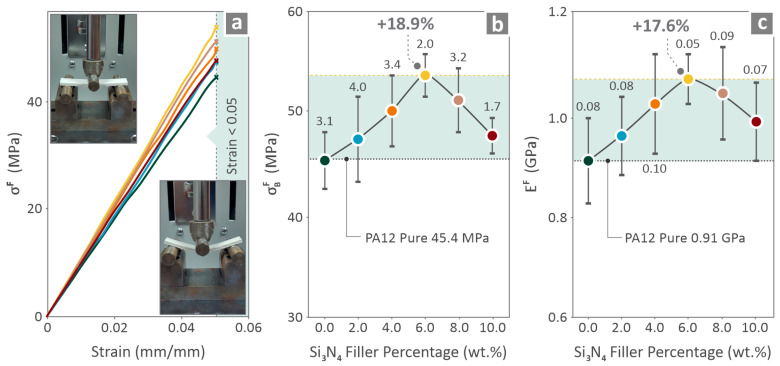
PA12/(0.0 wt. %–10.0 wt. %) Si_3_N_4_ flexural mechanical data: (**a**) stress vs. strain curves and flexural testing image; (**b**) strength vs. Si_3_N_4_ and (**c**) Young’s modulus vs. Si_3_N_4_. Different colors depict the different loadings in the nanocomposites.

**Figure 10:**
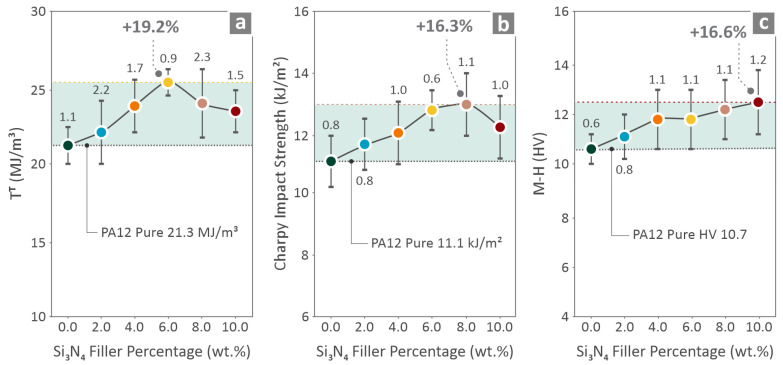
PA12/(0.0 wt. %–10.0 wt. %) Si_3_N_4_ data about the measured (**a**) tensile toughness, (**b**) impact strength (Charpy), and (**c**) microhardness. Different colors depict the different loadings in the nanocomposites.

For the presentation of the mechanical performance of the specimens, Figure 8, Figure 9 and Figure 10 were prepared, showing graphs that include data for PA12/(0.0 wt. %–10.0 wt. %) Si_3_N_4_ nanocomposites. Figure 8 shows the tension-related information in the stress vs. strain graph and testing images (Figure 8a), strength vs. filler percentage (Figure 8b), and modulus vs. filler percentage (Figure 8c). Among all the stress–strain curves, the one belonging to 6.0 wt. % composite appeared to reach higher stress levels. In addition, 6.0 wt. % was distinguished for its strength and modulus of elasticity levels, which revealed improvement by 23.9% and 17.1%, respectively, over pure PA12.

Figure 9 illustrates flexure-related information in the stress vs. strain graph and testing images (Figure 9a), strength vs. filler percentage (Figure 9b), and modulus vs. filler percentage (Figure 9c). Again, by observing the stress–strain curves, it is indicated that the one belonging to 6.0 wt. % nanocomposite reaches higher stress levels than the rest, achieving improvement by 18.9% and 17.6%, respectively, over pure PA12.

Figure 10a shows the tensile toughness results, 6.0 wt. % possesses the highest levels, being 19.2% increased over pure PA12. Figure 10b shows the Charpy impact strength levels, which are the highest in the case of 8.0 wt. %, by 16.3%. Figure 10c presents the M-H results distinguishing 10.0 wt. % as the one having the highest levels, by being 16.6% over pure PA12.

### 3.6. Structural Characteristics (Quality Metrics)

**Figure 11:**
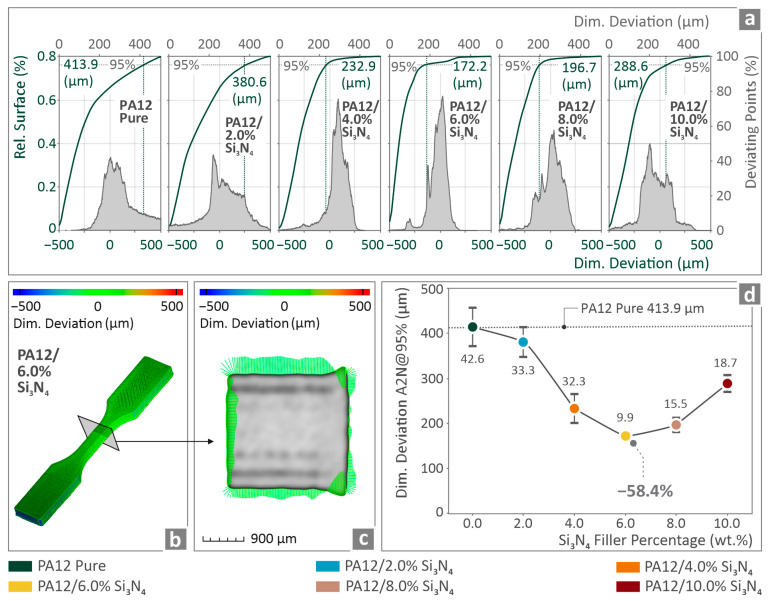
(**a**) PA12/(0.0 wt. %–10.0 wt. %) Si_3_N_4_ rel. surface and points deviating from nominal geometry vs. dimensional deviation graphs, (**b**,**c**) geometrical accuracy of PA12/6.0 wt. % Si_3_N_4_ tensile coupon via color coding mapping, and (**d**) geometrical accuracy vs. filler percentage graph considering all of the PA12/Si_3_N_4_ composites.

In Figure 11a, the dimensional difference findings are presented in the related surface and deviating points vs. dimensional accuracy graphs for all the PA12/Si_3_N_4_ nanocomposites. Figure 11b,c show the geometrical accuracy of PA12/6.0 wt. % Si_3_N_4_ tensile specimen via color coding mapping. Figure 11d illustrates the dimensional accuracy findings of all the PA12/Si_3_N_4_ nanocomposites vs. the filler percentage, indicating a drastic reduction in PA12/6.0 wt. % Si_3_N_4_ dimensional deviation by 58.4%, in relation to pure PA12.

**Figure 12:**
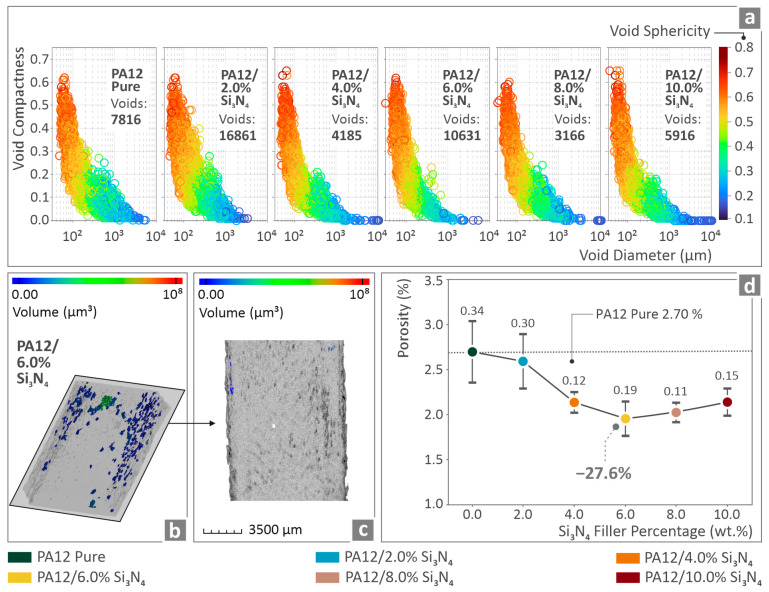
(**a**) PA12/(0.0 wt. %–10.0 wt. %) Si_3_N_4_ void sphericity and void compactness vs. void diameter graphs, (**b**,**c**) porosity of PA12/6.0 wt. % Si_3_N_4_ specimen via color coding mapping, and (**d**) porosity vs. filler percentage graph considering all of the PA12/Si_3_N_4_ composites.

Figure 12a shows the porosity results presented in the void sphericity and void compactness vs. void diameter graphs for all PA12/Si_3_N_4_ nanocomposites. Figure 12b,c show the porosity of PA12/6.0 wt. % Si_3_N_4_ specimen via color coding mapping. Figure 12d illustrates the porosity of all the PA12/Si_3_N_4_ nanocomposites vs. the filler percentage, revealing a reduction of 27.6% below pure PA12 for PA12/6.0 wt. % Si_3_N_4_.

### 3.7. Morphological Characteristics

**Figure 13:**
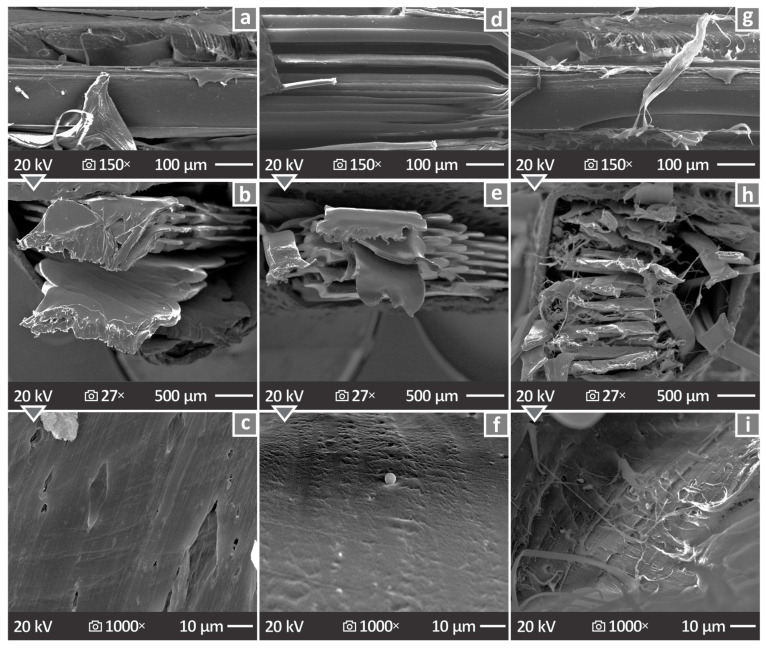
SEM illustrations of the side (lateral) surface at a magnification of 150× and fracture area at 27× and 1000× magnifications with regard to (**a**–**c**) PA12/2.0 wt. % Si_3_N_4_, (**d**–**f**) PA12/4.0 wt. % Si_3_N_4_, and (**g**–**i**) PA12/6.0 wt. % Si_3_N_4_.

**Figure 14:**
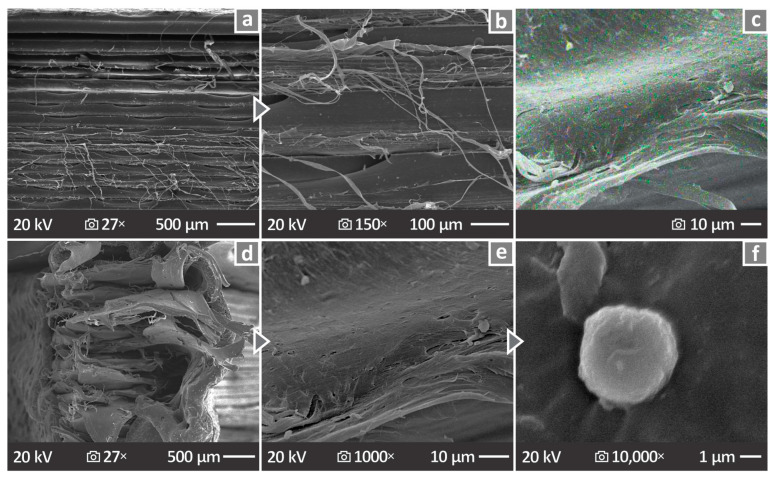
PA12/8.0 wt. % Si_3_N_4_ (**a**,**b**) SEM images of the lateral surface at 27× and 150× magnifications, (**c**) EDS mapping image presenting the distribution in the observation region of the Si element (nanoparticles dispersion), and (**d**–**f**) SEM images of the fracture surface at 27×, 1000×, and 10,000× magnifications.

The morphological characteristics of the specimens are presented in Figure 13 and Figure 14 (SEM images). Figure 13a,c refer to PA12/2.0 wt. % Si_3_N_4_, showing its side (lateral) surface at a magnification of 150× and fracture section at 27× and 1000× magnifications, respectively. The same is presented in Figure 13d–f for PA12/4.0 wt. % Si_3_N_4_ and in Figure 13g–i for PA12/6.0 wt. % Si_3_N_4_. The layering of the side surfaces appeared to be significantly uneven and not well distributed, whereas the fracture surface images indicated a remarkably ductile response in the specimens.

Figure 14a,b show the PA12/8.0 wt. % Si_3_N_4_ lateral surface images at 27× and 150× magnification, respectively, while in Figure 14c, there is an image of the same nanocomposite presenting the EDS extracted results about the nanomaterial dispersion (EDS mapping presenting the distribution in the observation area of the Si element). Figure 14d–f depict the fractured surface images of the same nanocomposite at 27×, 1000×, and 10,000× magnifications, respectively. In this case, the layering is more well-distributed, but voids also exist.

## 4. Discussion

The efficacy of silicon nitride as a reinforcement in PA12 within the context of MEX AM was evaluated in the current research through various characterization methods, which were applied to the produced nanocomposites. Preliminary tests were performed to determine the optimum printing conditions for the fabrication of the samples. The ±45 raster orientation was selected for the 3D-printed structure, as it contributes to reducing the anisotropy in the samples, as reported in the literature [111,112]. Regarding the nozzle temperature, preliminary tests showed that the 230 °C temperature achieved 3D printing results, with good layer bonding and without layer delamination issues, while the surface quality was improved. DSC showed that T_m_ was 175 °C; however, the experiments showed that a higher temperature was needed to improve the 3D printing results. This is expected since DSC T_m_ represents only the onset of crystalline melting under near-equilibrium conditions, whereas extrusion involves high shear, short residence times, and rapid heat losses [113,114]. The purpose of the present study was to analyze the effect of the filler in the matrix and not to investigate the effect of the printing parameters; therefore, the optimization of the printing parameters, such as the printing temperature, was deliberately not performed. Such research has been conducted by this group in the past, utilizing optimization models based on full factorial, Taguchi, and Box–Behnken designs, in which the effects of the filler, nozzle temperature, and bed temperature were investigated [115].

The thermal response of the nanocomposites depicted in Figure 4 suggests that the addition of nanoparticles to the polymeric matrix did not considerably affect the thermal stability of PA12. The temperature at which the mass started to drastically decompose in the TGA slightly increased with the addition of the nanopowder, indicating that the addition of the nanopowder increased the thermal stability of the PA12 thermoplastic. This can be attributed to the Si_3_N_4_ nanoparticles creating a physical barrier with the polymer, effectively slowing the process of thermal decomposition of the matrix [116]. Moreover, the addition of well-dispersed Si_3_N_4_ nanoparticles reduces chain scission because of their effects on the mobility [117]. The high thermal conductivity of Si_3_N_4_ nanoparticles allows the heat generated during thermal decomposition to be distributed over a large area, preventing large quantities of polymer from being degraded rapidly [118]. Furthermore, the TGA findings indicate that the residual mass increased almost linearly with an increase in the nanoparticle content in the matrix, which is the expected finding. In the DSC graphs, the addition of nanoparticles to the matrix did not considerably affect T_m_. Regardless of the filler content, it remained stable. Ceramic nanoparticles do not substantially change the crystal unit cell size or lamellar thickness, which preserves T_m_ [119,120].

The rheological characteristics of PA12 appeared to be modified by the introduction of Si_3_N_4_ filler, which increased the viscosity and decreased the MFR (Figure 6). The increase in the melt viscosity of PA12 due to the addition of Si_3_N_4_ nanoparticles occurs because of an increase in the interactions between the polymer and particles, as well as the creation of a polymer–nanoparticle network. The chains of the polymers become attracted to the surfaces of the nanoparticles, producing bridges and loops of chain segments that limit the movement of the chains, resulting in decreased mobility and relaxation time, and hence a higher zero-shear viscosity. The introduction of nanoparticles creates additional physical obstacles that impede the chain motion during diffusion. The polymer must travel through path sections between nanoparticles, which provide further entropic barriers, particularly at higher concentrations of loaded filler. Moreover, at sufficiently high filler levels, a percolated or semi-percolated network of fillers can be formed, providing additional resistance to molecular flow and resulting in a further increase in melt viscosity [121,122].

Herein, the viscosity increased up to 4 wt. % and then started to decrease (still higher than pure in all cases), while MFR constantly decreased with filler loading. At shear rates up to 10^−1^, a clear trend was observed with increasing viscosity up to 4.0 wt. %. At higher loadings, it decreased to the maximum loading tested. At shear rates higher than 10^−1^, the curves crossed. The 6.0 wt.% loading shows median viscosity among the nanocomposites, as the viscosity starts to decrease beyond the 4.0 wt. % loading. At low to moderate amounts of filler loading, Si_3_N_4_ nanoparticles are well dispersed, allowing polymer chains to adsorb onto particle surfaces, resulting in an increased hydrodynamic volume and increased inter-particle interactions, causing an increase in melt viscosity and therefore producing a low shear yield/elastic response [123]. Beyond 4 wt. %, particle clusters and non-homogeneously sized regions were visible, leading to a decrease in the available surface area on the fillers for immobilizing polymer chains, which causes the viscosity of the mixtures measured by oscillatory and rotational rheometry to decrease [124]. On the other hand, MFR is a single-point test performed at high shear and has limited application in characterizing die flow resistance. The MFR is also sensitive to melt elasticity and the presence of clusters of materials. If either agglomerates or melt elasticity increases, then MFR will typically decrease as mass throughput decreases; therefore, MFR will generally decrease monotonically, whereas both low-shear viscosity and mid-shear viscosity can show both decreasing and increasing (non-monotonic) trends [125].

The introduction of Si_3_N_4_ into the PA12 matrix was beneficial for the two quality metrics assessed using micro-CT. The dimensional accuracy improved by 58.4% in the nanocomposite with 6 wt. % Si_3_N_4_ content (Figure 11). At higher loadings, the dimensional deviation increases, maintaining values below those of the unfilled PA12 polymer. This can be explained by the reduction in the viscosity at higher loadings. This makes the flow of the material more difficult, thus affecting layer fusion and the overall formation of the 3D-printed structure. The presence of well-dispersed Si_3_N_4_ nanoparticles assisted in reducing the amount of polymer that underwent thermal contraction. Thus, shrinkage and warpage are reduced. In addition, the presence of these nanoparticles increases the overall stiffness of the bulk material, thereby improving not only strand stability but also dimensional accuracy [126]. When the loading exceeds 6 wt. %, particle agglomeration creates a localized separation between the different materials along with a lower quality bond strength between the layers in those areas (resulting in increased internal voids). These voids and associated stresses can negatively affect dimensional accuracy [127].

A similar trend was observed for porosity (Figure 12). This value decreased by 27.6% for the 6 wt. % nanocomposite and slightly increased up to the highest loading assessed. Again, its values were lower than those of the unfilled PA12 for all loadings assessed, showing that the addition of the Si_3_N_4_ nanoparticles was beneficial to the PA12 polymer for this quality metric. This non-monotonic pattern can be attributed to mechanisms similar to those mentioned above for the rheological properties. The literature also reports that specimens with lower porosity levels exhibit better mechanical properties [128,129]. This contributed to the highest mechanical response of the nanocomposites with 6 wt. % loading as well.

The side and fracture surfaces of the coupons were examined using SEM at different magnifications. The captured SEM images indicated unstable layering characterized by many defects, whereas the overall behavior of the specimens for mechanical testing was ductile (Figure 13). However, no major particle clustering was observed in the samples (Figure 14) through EDS mapping. Sufficient nanoparticle dispersion in the polymeric matrix was pursued through different efforts in four steps in the methodology followed. First, the raw material mix, which was intense enough to distribute the nanoparticles in the matrix as much as possible, then the use of a special extruder for material mixing to produce the filament, then the inspection of the samples with SEM and EDS for nanoparticle clustering location, and finally the study of the mechanical properties deviation, in which high values would denote differences in the composition of the samples. SEM was used to confirm the size and shape of the nanoparticles (Figure 2). The shape was irregular and prismatic, and its size was in agreement with the nominal value of 760 nm.

Mechanical tests were conducted on the filaments (Figure 7) and 3D-printed coupons (Figure 8, Figure 9 and Figure 10). The tensile strength and Young’s modulus for the 6.0 wt. % nanocomposite showed an improvement close to 20.0%, in relation to pure PA12. Overall, the filament values followed a similar pattern to the tensile test values on the 3D-printed samples, showing consistency in the results. The properties of the 6.0 wt. % nanocomposites for the tensile specimens revealed a 23.9% and 17.1% increase, respectively, and the flexural specimens showed an 18.9% and 17.6% increase, respectively, for the same 6.0 wt. % filler percentage. Higher filler percentages led to a reduction, but still improved their values in relation to pure PA12. Considering toughness, the same nanocomposite (6.0 wt. %) provided the highest levels, which were 19.2% higher than that of pure PA12. This can be explained by the increased strength and stiffness of the specific loading compared to those of the other nanocomposites assessed. The increased toughness values of the nanocomposites denote that the introduction of Si_3_N_4_ nanoparticles in the polymeric matrix improves the capability of the PA12 to absorb energy when subjected to loads. Furthermore, a 6.0 wt. % nanocomposite had the highest scores in the two quality metrics assessed (dimensional accuracy and porosity), showing a clear correlation between quality and mechanical performance in these nanocomposites. Better-quality built parts (higher geometrical accuracy and reduced 3D printing structure porosity) have an overall higher mechanical response. In higher-than-4.0 wt. % loadings, as analyzed above, viscosity started to increase in the nanocomposites, which has contributed to the decline of the quality and performance properties evaluated.

The inclusion of Si_3_N_4_ nanoparticles in MEX 3D-printed PA12 significantly improved its mechanical performance via various synergistic mechanisms. Rigid-filler nanoparticles enable efficient stress transfer from the polymer matrix because of their effectiveness as reinforcing fillers. By forming strong interfacial interactions between PA12 chains and the Si_3_N_4_ fillers, premature failure is reduced, thus load-bearing performance is improved. Furthermore, nanoparticles can reduce the porosity of the resultant material by improving melt flow characteristics and promoting layer-to-layer adhesion. Moreover, the literature reports that the reduction of porosity in 3D printing samples is beneficial for mechanical strength [21,130]. Therefore, the reduced porosity should also have contributed to the improved mechanical performance in this case, along with the introduction of nanoparticles. The literature on the porosity was also verified in this case.

Charpy impact strength and microhardness were the only properties that indicated their highest levels at different nanocomposite loadings, namely 8.0 wt. % and 10.0 wt. % respectively, by being 16.3% and 16.6% over pure PA12. The impact strength decreased by 10 wt. % loading. At 8.0 wt. % possible clustering of the particles may help absorb energy, thus maximizing the impact strength. As the filler content increased further (10 wt. %), larger particle clusters increased brittleness. A higher porosity contributes to the formation of interfacial defects, which act as stress concentrators, leading to a decrease in the impact strength [131]. The microhardness was higher in the nanocomposites with higher loading. This is attributed to the hard nature of the filler, which contributes to the resistance to plastic indentation [132].

**Figure 15:**
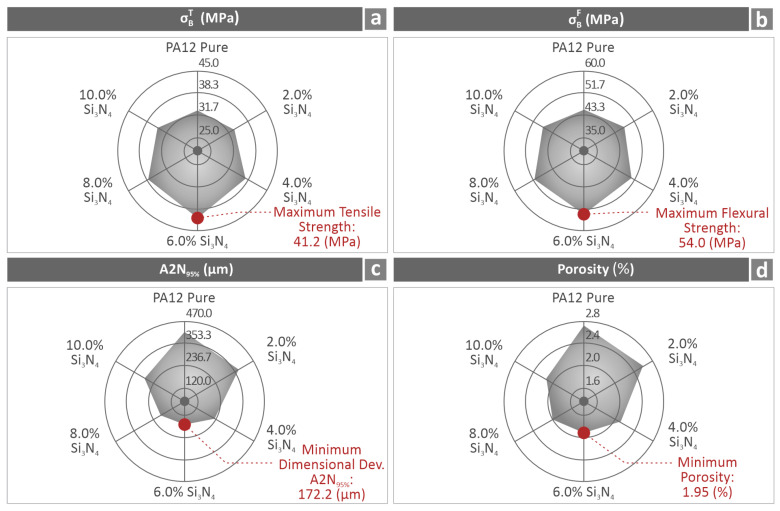
Spider-type graphs summarizing the findings of (**a**) tensile and (**b**) flexural strength, (**c**) geometrical accuracy, and (**d**) porosity of the assessed PA12/(0.0 wt. %–10.0 wt. %) Si_3_N_4_ specimens.

Figure 15 summarizes the main experimental findings. It is separated into four sections, each including a spider graph of the values belonging to four of the measured metrics of the PA12/Si_3_N_4_ 3D-printed coupons. The displayed properties are the tensile strength (Figure 15a), flexural strength (Figure 15b), dimensional deviation (geometrical accuracy) (Figure 15c), and porosity (Figure 15d). The maximum and minimum values measured for each property are highlighted.

## 5. Conclusions

Herein, the reinforcement efficacy of Si_3_N_4_ nanopowder on the popular PA12 thermoplastic was evaluated. The aim was to introduce novel nanocomposites in MEX AM featuring improved mechanical performance and overall properties (e.g., print quality) by utilizing PA12 as the matrix material. Nanocomposite mixtures of PA12/Si_3_N_4_ were formulated and extruded into filaments, which were subsequently employed for 3D printing the samples. The mixtures were prepared with filler loadings in the range of 2.0 wt. %–10.0 wt. %. Nanocomposites were evaluated using various characterization techniques, that is, mechanical, thermal, structural, morphological, and chemical. Mechanical testing was conducted on both the filaments and 3D-printed specimens. The thermal characterization and rheological properties of the samples were evaluated with respect to porosity and dimensional deviation using micro-CT scanning. The findings were:

- PA12/6.0 wt. % Si_3_N_4_ samples indicated the maximal improvement in mechanical properties compared to pure PA12.
- The tensile strength, Young’s modulus, flexural strength, and stiffness were improved by 23.9%, 17.1%, 18.9%, and 17.6%, respectively.
- The impact strength improved by 16.3% on PA12/8.0 wt. % Si_3_N_4_, while microhardness improved 16.6% on the samples with 10.0 filler content.
- PA12/6.0 wt. % Si_3_N_4_ samples also showed the highest dimensional accuracy (58.4% improvement) and the lowest porosity (27.6% reduced), proving for the specific nanocomposites a clear correlation between high mechanical performance and print quality.

Overall, this hypothesis was confirmed, and the nanocomposites demonstrated considerable potential for new applications, expanding the usability of the popular PA12 in MEX AM. Future work can focus on additional mechanical tests, such as thermomechanical tests or testing under high-speed and dynamic loading conditions. Furthermore, efforts can be made to locate the optimum 3D printing parameters, the exact saturation threshold, and the potential for the industrialization of the method.

## Figures and Tables

**Table 1:** Reinforcing effect of Si_3_N_4_ nanoparticles on different thermoplastics in 3D printing (MEX and vat photopolymerization methods are considered).

Increase (%)	PA12	PLA [84]	(PP) [85]	PETG [86]	HDPE [87]	ASA [89]	ABS [90]	Biomed Resin [88]
Tensile strength	23.9	40.4	16.0	24.5	21.0	11.6	25.6	23.6
Flex. strength	18.9	68.0	15.7	16.6	20.6	5.7	29.4	44.8
Opt. loading	6.0	6.0	2.0	6.0	6.0	2.0	6.0	1.0

**Table 2:** DSC findings: ($X_{c}\;\left(\%\right)$: crystallinity, $\Delta H_{m}\;\left(J/g\right)$: melting enthalpy, $w\;\left(g\right)$: PA12 mass, and $\Delta H_{m}^{0}\;\left(J/g\right)$: theoretical heat of fusion for 100% crystalline PA12) [100,101].

Composition	T_m_ (°C)	Δ*H_m_* (*J*/*g*)	*w* (*g*, PA12)	*X_c_* (%)
PA12 Pure	179.9	57.6	1.00	27.5
PA12 vs. Si_3_N_4_ 2.0 wt. %	179.2	44.3	0.98	21.6
PA12 vs. Si_3_N_4_ 4.0 wt. %	179.5	42.1	0.96	21.0
PA12 vs. Si_3_N_4_ 6.0 wt. %	179.8	42.3	0.94	21.5
PA12 vs. Si_3_N_4_ 8.0 wt. %	179.7	41.1	0.92	21.4
PA12 vs. Si_3_N_4_ 10.0 wt. %	179.7	37.4	0.90	19.9

**Table 3:** Major Raman peak variations in PA12/Si_3_N_4_ nanocomposites from unfilled PA12.

1108	Peak drop	Medium change in C-O-C stretching [102]
1113	Peak drop	Medium change in C-O-C stretching [102]
1296	Change	Medium change in C-O-C stretching [102]
1436	Change	Medium change in CH_2_ deformation [102,105]
2844	Peak drop	Large change in CH_2_ symmetric vibration [103]
2844–2874	Peak drop	Large change in CH_2_ symmetric vibration [103]
2886–2948	Peak drop	Large change in a range of methylation vibration modes [103]

## Data Availability

The authors confirm that data supporting the findings of this study are available within the article and its Supplementary Materials.

## Supplementary Materials

The following supporting information can be downloaded from https://www.mdpi.com/article/10.3390/nano16010047/s1.
