# Supplementary material for: Optimization of Silicon Nitride Nanopowder Content in Polyamide 12 (PA12) in Extrusion-Based Additive Manufacturing
Source: Nanomaterials (Basel). 2025 Dec 29;16(1):47. doi: 10.3390/nano16010047 (PMC12787588; doi:10.3390/nano16010047)
Supplement: Supplementary file 1 [file nanomaterials-16-00047-s001.zip › nanomaterials-4039367-supplementary.pdf]

nanomaterials

MDPI

Article

Optimization of silicon nitride nanopowder content in polyamide 12 (PA12) in extrusion-based additive manufacturing

Markos Petousis [1], Apostolos Korlos [2], Nikolaos Michailidis [3,4], Vassilis Papadakis [5], Apostolos Argyros [3,4], Nikolaos Mountakis [1], Maria Spyridaki [1], Athena Maniadi [1,6], Amalia Moutsopoulou [1] and Nectarios Vidakis [1,*]

[1] Department of Mechanical Engineering, Hellenic Mediterranean University, Heraklion 71410, Greece, markospetousis@hmu.gr; mountakis@hmu.gr; mspyridaki@hmu.gr; amalia@hmu.gr, vidakis@hmu.gr;
[2] Department of Industrial Engineering and Management, International Hellenic University, 14th km, Thessaloniki—N. Moudania, Thermi, 57001 Thessaloniki, Greece; apkorlos@ihu.gr
[3] Physical Metallurgy Laboratory, Mechanical Engineering Department, School of Engineering, Aristotle University of Thessaloniki, 54124 Thessaloniki, Greece, nmichail@auth.gr; aargyros@auth.gr
[4] Centre for Research & Development of Advanced Materials (CERDAM), Center for Interdisciplinary Research and Innovation, Balkan Centre, Building B', 10th km Thessaloniki-Thermi road, 57001, Thessaloniki, Greece
[5] Department of Industrial Design and Production Engineering, University of West Attica, 122 43 Athens, Greece, v.papadakis@uniwa.gr
[6] Department of Materials Science and Technology, University of Crete, 70013 Heraklion Crete, Greece; maniadi@materials.uoc.gr
* Corresponding author: Nectarios Vidakis, e-mail: vidakis@hmu.gr, Tel.: +302810379227

Abstract

The use of polyamide-12 (PA12) thermoplastics in additive manufacturing (AM) is promising owing to their mechanical properties and printability. However, in load-bearing applications, improvements in mechanical strength and stiffness are sought after. Herein, the reinforcement efficiency of silicon nitride (Si_3N_4) nanoparticles in the PA12 matrix was explored. The filler loading varied between 2.0 wt. % and 10.0 wt. %. The nanocomposites were extruded into filament using melt compounding for subsequent material extrusion (MEX) 3D printing. PA12/ Si_3N_4 nanocomposites were examined for their thermal, rheological, morphological, and structural characteristics. For mechanical characterization, flexural, tensile, microhardness, and Charpy impact data were obtained. For structural examination, porosity and dimensional deviation were assessed. Scanning electron microscopy (SEM) was used to investigate morphology and chemical composition. The results indicate that Si_3N_4 nanopowder significantly improved all mechanical properties, with a greater than 20% increase in tensile strength and elastic modulus when compared to neat PA12. The structural characteristics were also improved. These findings indicate that Si_3N_4 nanoparticles provide a viable reinforcement filler for PA12 for use in lightweight, robust structural components fabricated using MEX AM. Furthermore, it can be stated that ceramic-polymer nanocomposites further improve the applicability of PA12, where high mechanical performance is required.

Keywords: Polyamide 12 (PA12); Silicon Nitride (Si_3N_4); Additive Manufacturing (AM); Three-Dimensional (3D) Printing; nanocomposites; ceramics;

Academic Editor: Wei Zhang

Received: 26 November 2025
Revised: 13 December 2025
Accepted: 25 December 2025
Published: 29 December 2025

Copyright: © 2025 by the authors. Submitted for possible open access publication under the terms and conditions of the Creative Commons Attribution (CC BY) license.

S1. Raman Spectroscopy

The Raman spectra were acquired utilizing a LabRAM HR Raman Spectrometer from HORIBA Scientific, based in Kyoto, Japan. A 532 nm solid-state laser module, capable of delivering a maximum power of 90 mW, was employed for excitation. The spectral resolution of the Raman spectrometer was approximately 2 cm^{-1}, achieved with a grating featuring 600 grooves. An Olympus objective lens (LMPlanFL N) with a numerical aperture of 0.5 was used to focus light onto the sample and collect the Raman signals. This objective lens, with a 50× magnification, had a working distance of 10.6 mm. A Neutral Density filter with 10% transmittance was utilized to limit the laser power, which was measured at 4 mW for the sample. The measurement volume was determined to be 1.7 μm laterally and 2 μm axially. The Raman spectra were recorded in the range of 40 to 3900 cm^{-1}, achieved through three optical windows. Each measurement point had an exposure time of 4 seconds, with five accumulations. The irradiated areas were visually inspected to ensure no discoloration or degradation occurred due to laser exposure.

The raw Raman data were processed using LabSpec software from HORIBA in Kyoto, Japan. Each spectrum was processed using the same method: a) removal of cosmic rays; b) signal denoising with a 5-point kernel; c) data cropping between 800 and 3200 cm^{-1}; d) background removal using a 6th-degree polynomial; e) normalization of data by the maximum value. The Raman peaks of the unfilled PA12 coupon were detected and assessed with the bibliography and are depicted in Table S1.

Table S1. Major Raman peaks of pure PA12 were identified and their related assignments.

Wavenumber (cm^{-1})	Raman peak assignment
1000	C-H in-plane bending [1]
1029	C–C and C-O vibration [2]
1062	C-O-C stretching [1]
1106	C-O-C stretching [1]
1153	Skeletal deformation [2]
1193	C-O-C stretch [3]
1294	C-O-C stretching [1]
1435	C-H_2 deformation [1,4]
1581	unidentified
1600	Skeletal vibration of the C=C aromatic ring [5,6]
1632	C=C vibration [7]
2849	C-H_2 symmetric stretching [2]
2883	C-H_2 symmetric stretching [2] [8]
2899	C-H stretching [1]
2922	C-H_2 asymmetric stretching [2]

S2. Thermal characterization

TGA and DSC were selected for the thermal characterization of PA12/ Si_3N_4 nanocomposites. TGA data were obtained via the utilization of a Diamond Perkin Elmer (Massachusetts, USA) apparatus (temperature cycle of 40–550 °C, increase rate of 10 °C / min). DSC was performed using a Discovery Series DSC-25 DSC calorimeter (TA Instruments, Delaware, United States) (featuring an RSC-90 Refrigerated Cooling System). An inert environment and high-purity N_2 (nitrogen gas) prevailed during both the TGA and DSC.

In DSC, crystallinity Xc was calculated utilizing the following formula:

$$X_c(\%) = \left(\frac{\Delta H_m}{w \cdot \Delta H_m^0}\right) \times 100 \qquad \text{(S1)}$$

Where:

X_c (%) → crystallinity

ΔH_m (J/g) → melting enthalpy

https://doi.org/10.3390/nano16010047

$w\ (g)$ → PA12 mass

$\Delta H_m^0\ (J/g)$ → theoretical heat of fusion for 100% crystalline PA12. This is a commonly used literature constant for calculating crystallinity from DSC. It was considered as $\Delta H_m^0 = 209.3(J/g)$, with the value taken from literature [9,10].

S3. Rheometric Characterization

Rheometric characteristics were revealed with the assistance of a DHR-20 Discovery Hybrid Rotational Rheometer (TA Instruments, Delaware, USA) (ASTM D1238-13, for MFR), featuring an Environmental Test Chamber with a parallel-plate setup (temperature regulation). Measured point underwent 10 seconds of acquisition, with the aim of preventing excessive heating, as well as decomposition. The material flow rate values at specific temperatures and pre-selected pressures were obtained through both MFR and rotational rheometric tests.

S4. Structural Characterization

Characteristics related to the structure of the specimens, namely porosity and dimensional deviation, were revealed using micro-computed tomography (μ-CT) technology. A Tomoscope HV Compact 225 kV Micro Focus CT-scanner (Werth Messtechnik GmbH, Giessen, Germany) was utilized, featuring a 1024 × 1024-pixel sensor. VG Studio MAX 2.2 software (Volume Graphics GmbH, Heidelberg, Germany) was employed for data analysis. For the measurement of dimensional accuracy, a 75 L setup, 72.58 μm resolution on X-axis, and 72.65 μm resolution on Y-axis were utilized, while for the porosity, there was a 16 L setup, 15.46 μm resolution on X-axis, and 15.49 μm resolution on Y-axis. In both cases, there were 1600 sections per revolution.

References

1. Stuart, B.H. Temperature Studies of Polycarbonate Using Fourier Transform Raman Spectroscopy. *Polymer Bulletin* **1996**, *36*, 341–346, doi:10.1007/BF00319235.
2. Makarem, M.; Lee, C.M.; Kafle, K.; Huang, S.; Chae, I.; Yang, H.; Kubicki, J.D.; Kim, S.H. Probing Cellulose Structures with Vibrational Spectroscopy. *Cellulose* **2019**, *26*, 35–79.
3. Resta, V.; Quarta, G.; Lomascolo, M.; Maruccio, L.; Calcagnile, L. Raman and Photoluminescence Spectroscopy of Polycarbonate Matrices Irradiated with Different Energy 28Si+ Ions. *Vacuum* **2015**, *116*, 82–89.
4. Zimmerer, C.; Matulaitiene, I.; Niaura, G.; Reuter, U.; Janke, A.; Boldt, R.; Sablinskas, V.; Steiner, G. Nondestructive Characterization of the Polycarbonate-Octadecylamine Interface by Surface Enhanced Raman Spectroscopy. *Polymer Testing* **2019**, *73*, 152–158.
5. Luiz, B.K.; Amboni, R.D.; Prates, L.H.M.; Bertolino, J.R.; Pires, A.T. Influence of Drinks on Resin Composite: Evaluation of Degree of Cure and Color Change Parameters. *Polymer testing* **2007**, *26*, 438–444.
6. Gatin, E.; Iordache, S.-M.; Matei, E.; Luculescu, C.-R.; Iordache, A.-M.; Grigorescu, C.E.A.; Ilici, R.R. Raman Spectroscopy as Spectral Tool for Assessing the Degree of Conversion after Curing of Two Resin-Based Materials Used in Restorative Dentistry. *Diagnostics* **2022**, *12*, 1993.
7. Peris-Díaz, M.D.; Łydżba-Kopczyńska, B.; Sentandreu, E. Raman Spectroscopy Coupled to Chemometrics to Discriminate Provenance and Geological Age of Amber. *Journal of Raman Spectroscopy* **2018**, *49*, 842–851.
8. Liu, X.; Zou, Y.; Li, W.; Cao, G.; Chen, W. Kinetics of Thermo-Oxidative and Thermal Degradation of Poly (d, l-Lactide)(PDLLA) at Processing Temperature. *Polymer Degradation and Stability* **2006**, *91*, 3259–3265.
9. Mark, J.E. *Polymer Data Handbook*; Oxford University Press New York, NY, 2009; ISBN 978-0-19-518101-2.
10. Martienssen, W.; Warlimont, H. *Springer Handbook of Condensed Matter and Materials Data*; Springer Science & Business Media, 2006;

Disclaimer/Publisher's Note: The statements, opinions and data contained in all publications are solely those of the individual author(s) and contributor(s) and not of MDPI and/or the editor(s). MDPI and/or the editor(s) disclaim responsibility for any injury to people or property resulting from any ideas, methods, instructions or products referred to in the content.

https://doi.org/10.3390/nano16010047
